# Beyond Clinical High-Risk State for Psychosis: The Network Structure of Multidimensional Psychosis Liability in Adolescents

**DOI:** 10.3389/fpsyt.2019.00967

**Published:** 2020-02-11

**Authors:** Eduardo Fonseca-Pedrero, Javier Ortuño-Sierra, Felix Inchausti, Juan Francisco Rodríguez-Testal, Martin Debbané

**Affiliations:** ^1^ Department of Educational Sciences, University of La Rioja, Logroño, Spain; ^2^ Centro de Investigación Biomédica en Red de Salud Mental (CIBERSAM), Oviedo, Spain; ^3^ Programa Riojano de Investigación en Salud Mental (PRISMA), Logroño, Spain; ^4^ Department of Mental Health, Servicio Riojano de Salud, Logroño, Spain; ^5^ Department of Psychology, University of Seville, Seville, Spain; ^6^ Faculty of Psychology and Educational Sciences, University of Geneva, Geneva, Switzerland; ^7^ Department of Clinical, Educational and Health Psychology, University College London, London, United Kingdom

**Keywords:** clinical high risk, schizotypal, schizotypy, network, complex dynamic system

## Abstract

**Objectives:**

The main goal of the present study was to analyze the network structure of schizotypy dimensions in a representative sample of adolescents from the general population. Moreover, the network structure between schizotypy, mental health difficulties, subjective well-being, bipolar-like experiences, suicide ideation and behavior, psychotic-like experiences, positive and negative affect, prosocial behavior, and IQ was analyzed.

**Method:**

The study was conducted in a sample of 1,506 students selected by stratified random cluster sampling. The Oviedo Schizotypy Assessment Questionnaire, the Personal Wellbeing Index–School Children, the Paykel Suicide Scale, the Mood Disorder Questionnaire, the Strengths and Difficulties Questionnaire, the Prodromal Questionnaire–Brief, the Positive and Negative Affect Schedule for Children Shortened Version, and the Matrix Reasoning Test were used.

**Results:**

The estimated schizotypy network was interconnected. The most central nodes in terms of standardized Expected Influence (EI) were ‘unusual perceptual experiences’ and ‘paranoid ideation’. Predictability ranged from 8.7% (‘physical anhedonia’) to 52.7% (‘unusual perceptual experiences’). The average predictability was 36.27%, implying that substantial variability remained unexplained. For the multidimensional psychosis liability network predictability values ranged from 9% (estimated IQ) to 74.90% (‘psychotic-like experiences’). The average predictability was 43.46%. The results of the stability and accuracy analysis indicated that all networks were accurately estimated.

**Conclusions:**

The present paper points to the value of conceptualizing psychosis liability as a dynamic complex system of interacting cognitive, emotional, behavioral, and affective characteristics. In addition, provide new insights into the nature of the relationships between schizotypy, as index of psychosis liability, and the role played by risk and protective factors.

## Introduction

The leitmotiv of psychosis high-risk paradigms [i.e., psychometric, genetic and Clinical High Risk (CHR)] is based on the ability to identify those individuals potentially at risk of developing psychosis in order to conduct prevention and prophylactic interventions (1, 2). Psychosis high risk approaches attempt to capturing early clinical (micro) phenotypes at early stages before care is needed and disability ensues. With these objectives in mind, proliferation of programs and centers specialized in early intervention in psychosis have emerged in the last twenty years (3–5). However, the “ultra-high risk” concept and “transition” paradigm have been questioned (6).

Psychosis high risk approaches assume (explicitly or implicitly) the idea of psychosis liability continuum (7). The construct that harbors the latent liability for schizophrenia and related manifestations is called schizotypy (8). At the phenotypic level, schizotypy can manifest itself, in a range variety of expressions, such us schizotypal traits, psychotic-like experiences, subclinical psychotic symptoms (i.e., CHR), frank psychotic symptoms, schizotypal personality disorder, or psychosis-spectrum disorders (2, 9). At population level, the non-clinical (or “soft”) expression of psychosis phenotype may represent the behavioral manifestation of risk for psychosis (7, 10–12) and psychopathology. In fact, schizotypy probably represents the most clearly tractable risk factor for schizophrenia spectrum disorders (13). In its structure, schizotypy is a multidimensional construct, composed basically of three factors (Cognitive-Perceptual, Negative, and Disorganization), which is consistent with the factor structure found in patients with psychosis and CHR samples (14–16).

Modern approaches of psychosis promote a developmental, staging, and transdiagnostic approach which takes into account the different dimensions of risk, as well as protective factors, that influence liability to psychopathology (3, 6, 17, 18). In addition, clinical and subclinical psychosis phenotypes can be seen as complex dynamic systems of interacting cognitive, emotional, behavioral, social, and affective traits (19, 20). This viewpoint, named network model, represents a recent theoretical approach in the psycho(patho)logy arena, although it is not new in the scientific field (21–23). Basically, the network model is evolving as a response to the biomedical model, which is being disseminated by the leading nosological systems (e.g., DSM and ICD). Thus, new psychopathological and psychometric approaches, like network framework or chaos theory (24), might provide new insights in psychosis and mental health fields. In addition, a dynamic approach of psychopathology can complement and give new insight to a traditionally DSM categorical viewpoint.

From network approach, mental disorders, like psychosis, can be seen as emergent properties that arise from mutual interactions between mental states (or symptoms, signs, traits, etc.) (25–29).These findings can be considered within the network model of onset of psychotic disorders proposed by Linscott and van Os (30). The onset for the outcome of these mental health problems can be understood in part as: a) different psychotic-like experiences and schizotypal traits (e.g., psychosis proneness) that causally impact on each other over time (within phenotype domain), becoming persistent and leading then to clinical impairment, and b) many factors from multiple levels of analysis (e.g., trauma, cannabis, bullying, genetic background, brain function, etc.) that also causally impact on each other over time within and across - vertically and horizontally- domains in psychosis expression (31).

A wide variety of issues still remain to be resolved in psychosis research. It is necessary to gain a deeper understanding of the nature and structure of multidimensional psychosis liability beyond diagnostic systems (based on macrophenotypes) and in early stages of developmental disorders. At the same time, it would be interesting considering both risk (i.e. suicide ideation, emotional problems) and protective factors (i.e. well-being, positive affect) in the individual, as dynamic complex systems. Overall, these studies might be relevant in order to improve our knowledge about etiological mechanisms as well as early detection and intervention strategies in mental health. In addition, network model provides an informative way to describe the complex relationships between a set of key variables, focusing on the local interactions at the level of smaller units that compose the psychological problems, such as emotional and behaviors manifestations, and not at the disorder level. Based on this developmental, staging, non-clinical, and transdiagnostic approach, adolescence is a relevant developmental period where many changes at bio-psycho-social level take place. Therefore, it becomes a crucial stage to identify and intercept the unfolding of mental health problems. Moreover, if it is considered that almost 75% of all mental disorders begin in the first two decades of life and many of these individuals start with subclinical phenomena and/or report prodromal symptoms before to clinical outcome (32, 33).

To date, there has been no in-depth examination about the network structure of schizotypy and its relationship with cognitive, emotional, social, and behavioral indicators. Interestingly, no previous studies have analyzed the role of protective factors, such as personal well-being, prosocial behavior, or positive affect in psychosis liability network. Within this research framework, the main goals of the present study were: a) To analyze the network structure of schizotypy dimensions (within domain), as indirect indicator of psychosis liability in a representative sample of adolescents from the general population; and b) To estimate the network structure of schizotypy dimensions, mental health difficulties, subjective well-being, bipolar-like experiences, suicide ideation and behavior, psychotic-like experiences, positive and negative affect, prosocial behavior, and IQ (between domains).

## Methods

### Participants

Stratified random cluster sampling was conducted at the classroom level, in an approximate population of 15,000 students selected from a region located in northern Spain. The students belonged to different public and concerted Educational Centers of Compulsory Secondary Education and Vocational Training, as well as to different socio-economic levels. The layers were created as a function of the geographical zone and the educational stage.

The initial sample consisted of 1,881 students, eliminating those participants who presented a high score in the Oviedo Infrequency Response Scale (more than 3 points) (n = 104), an age older than 19 (n = 170) or did not complete the tests or the neurocognitive battery (n = 101). A total of 1506 students, 667 men (44.3%), belonging to 34 schools and 98 classrooms participated in the study. The mean age was 16.1 years (SD = 1.36), ranging from age 14 to 19 years.

Nationality distribution of the participants was as follows: 89.9% Spanish, 3.7% Latin American (Bolivia, Argentina, Colombia, and Ecuador), 2.4% Romanian, 1% Moroccan, 0.7% Portuguese, 0.7% Pakistani, and 2% other nationalities.

### Instruments

*The Oviedo Schizotypy Assessment Questionnaire-Revised* (ESQUIZO-Qr) (34). The ESQUIZO-Qr is a self-report measure developed for the assessment of schizotypal traits in adolescents. It comprises a total of 62 items with Likert type response format in five categories (from 1 “totally disagree” to 5 “totally agree”). Its 10 subscales are derived empirically by means of factor analysis, which in turn are grouped into three general dimensions: Reality Distortion (e.g., Ideas of Reference, Magical Thinking, Unusual Perceptual Experiences, and Paranoid Ideation), Anhedonia (Physical Anhedonia and Social Anhedonia), and Social Disorganization (Odd Thinking and Speech, Odd Behavior, Lack of Close Friends, and Excessive Social Anxiety). In this revised version new items of Anhedonia dimension were added. Internal consistency levels for the subscales ranged from 0.62 to 0.90 and several sources of validity evidence with other psychopathology measures were gathered (34).

*The Personal Wellbeing Index- School Children* (PWI-SC) (35). The PWI-SC contains eight items of satisfaction, corresponding to different quality of life domains: standard of living, personal health, achievement in life, personal relationships, personal safety, feeling part of the community and future security. The PWI-SW has been validated in Spanish samples of adolescents (36). In the present study, the internal consistency, estimated with Cronbach’s alpha, was 0.81.

*The Paykel Suicide Scale* (PSS) (37). The PSS is a self-report tool designed for the evaluation of suicidal ideation and behavior (lifetime prevalence). It consists of a total of 5 items with a dichotomous response system Yes/No (score, 1 and 0, respectively). The scores range from 0 to 5. The Spanish adaptation of the PSS has demonstrated adequate psychometric properties (38, 39). In the present study, the internal consistency, estimated with Cronbach’s alpha, was 0.90.

*The Mood Disorder Questionnaire* (MDQ) (40). The MDQ consists of 13 yes/no items based on the DSM-IV criteria for bipolar disorder. A result is considered positive if the participant replies affirmatively to 7 or more items of the 13 proposed and if, in addition, the symptoms described occurred during the same time period (Criterion 2) and represented moderate or severe problems (Criterion 3). The Spanish version of the MDQ has demonstrated adequate psychometric properties (41). In the present study, the internal consistency, estimated with Cronbach’s alpha, was 0.85.

*The Strengths and Difficulties Questionnaire* (SDQ) (42). The SDQ is a self-report tool that is widely used for the assessment of different emotional and behavioral problems related to mental health in adolescents. The SDQ is made up of a total of 25 statements distributed across five subscales: Emotional symptoms, Conduct problems, Hyperactivity, Peer problems, and Prosocial behavior. In this study we used a Likert-type response format with three options (0 = “Not true”, 1 = “Somewhat true”, 2 = “Certainly true”). The Spanish version of the SDQ was used (43) (see https://www.sdqinfo.com/a0.html). In the present study, internal consistency levels for the SDQ subscales ranged from 0.72 to 0.87.

*The Prodromal Questionnaire–Brief* (PQ-B) (44). The PQ-B is a psychosis-risk screening measure containing 21-items that are answered in a dichotomous response format (true/false). The PQ-B asks additional questions regarding frequency/severity of impairment and distress, rated on a Likert-type (1 “strongly disagree” to 5 “strongly agree”). The Spanish validation of the PQ-B has demonstrated adequate psychometric properties (45). In the present study, the internal consistency of PQ-B total score, estimated with Cronbach’s alpha, was 0.89.

*The 10-Item Positive and Negative Affect Schedule for Children Shortened Version* (46). The PANAS-10, is a self-reported adjective checklist that contains two 5-item subscales designed to measure positive (i.e., joyful, cheerful, happy, lively, proud) and negative affect (i.e., miserable, mad, afraid, scared, sad). The PANAS-10 uses a Likert-type scale (ranging from 1, *very slightly or not at all*, to 5, *extremely or very much*). Evidences of internal consistency of the PANAS in Spanish population range from 0.86 to 0.90 for positive affect, and from 0.84 to 0.87 for negative affect (47). In the present study, internal consistency values for the PANAS ranged from 0.84 to 0.89.

*The Penn Matrix Reasoning Test* (PMRT) (48, 49). This is a task of the Penn Computerized Neurocognitive Battery-Child version developed to measure non-verbal reasoning within complex cognition domain. This task is composed by 20 items that can be considered as estimated IQ. The battery includes different neurobehavioral tasks adapted to youth samples that have demonstrated adequate psychometric properties (48, 49).

*The Oviedo Infrequency Scale* (INF-OV) (50). INF-OV was administered to the participants to detect those who responded in a random, pseudorandom or dishonest manner. The INF-OV instrument is a self-report composed of 12 items in a 5-point Likert- scale format (1 = completely disagree; 5 = completely agree). Students with more than three incorrect responses were eliminated from the present study.

### Procedure

The research was approved by the Educational Government of La Rioja and the Ethical Committee of Clinical Research of La Rioja (CEICLAR). The self-reports and neurocognitive battery were administered collectively through personal computers in groups of 10 to 30 students during normal school hours, and in a classroom specially prepared for this purpose. Administration took place under the supervision of researchers previously trained in a standard protocol. No incentive was provided for their participation. For participants under 18, parents were asked to provide a written informed consent in order for their child to participate in the study. Participants were informed about the confidentiality of their responses and the voluntary nature of the study.

### Data Analyses

#### General Network Estimation

The details of network analysis were documented in-depth elsewhere (51, 52). Two networks were estimated. First, within schizotypy dimensions. Second, between schizotypy, mental health difficulties, subjective well-being, bipolar-like experiences, suicide behaviors, psychotic-like experiences, positive and negative affect, prosocial behavior, and estimated IQ.

A network consists of nodes (e.g., ESQUIZO-Qr domains) and edges (unknown statistical relationships between nodes that need to be estimated). For the domains, which were constructed by summing items per domain and then standardizing the resulting variable, we estimated a Gaussian Graphical Model (GGM) (53). This model resulted in conditional dependence relations which are akin to partial correlations: if two nodes are connected in the resulting graph *via* an edge, they are statistically related after controlling for all other variables in the network; if they are unconnected, they are conditionally independent. For the layout, the Fruchterman-Reingold algorithm was used, placing the strongly connected nodes closer to each other and the least connected nodes far apart (51).

#### Network Inference

Concordantly to previous studies examining network (54), we estimated two measures: Expected Influence (EI) and predictability.

- EI is the sum of all edges of a node (55). We use EI instead of strength centrality (56), that has been used in prior works, because strength centrality uses the sum of absolute weights (i.e. negative edges are turned into positive edges before summing), which distorts the interpretation if negative edges are present.
- Predictability is an absolute measure of interconnectedness: it provides us with the variance of each node that is explained by all its neighbors (57). Predictability can be understood as an upper bound of controllability: assuming that all undirected edges connected to a node point towards this node, predictability quantifies how much impact neighbors have on a focal node by intervening on them. In the figures, dark areas in the circle around nodes can be interpreted akin to R^2^ (% of explained variance) (57).

#### Network Stability

To test network stability and accuracy, we used bootstrapping routines implemented in the R-package *bootnet* (58).

SPSS 22.0 (59), *R* (60), and FACTOR (61) were used for these analyses.

## Results

### Network Structure of Schizotypy

**Figure 1:**
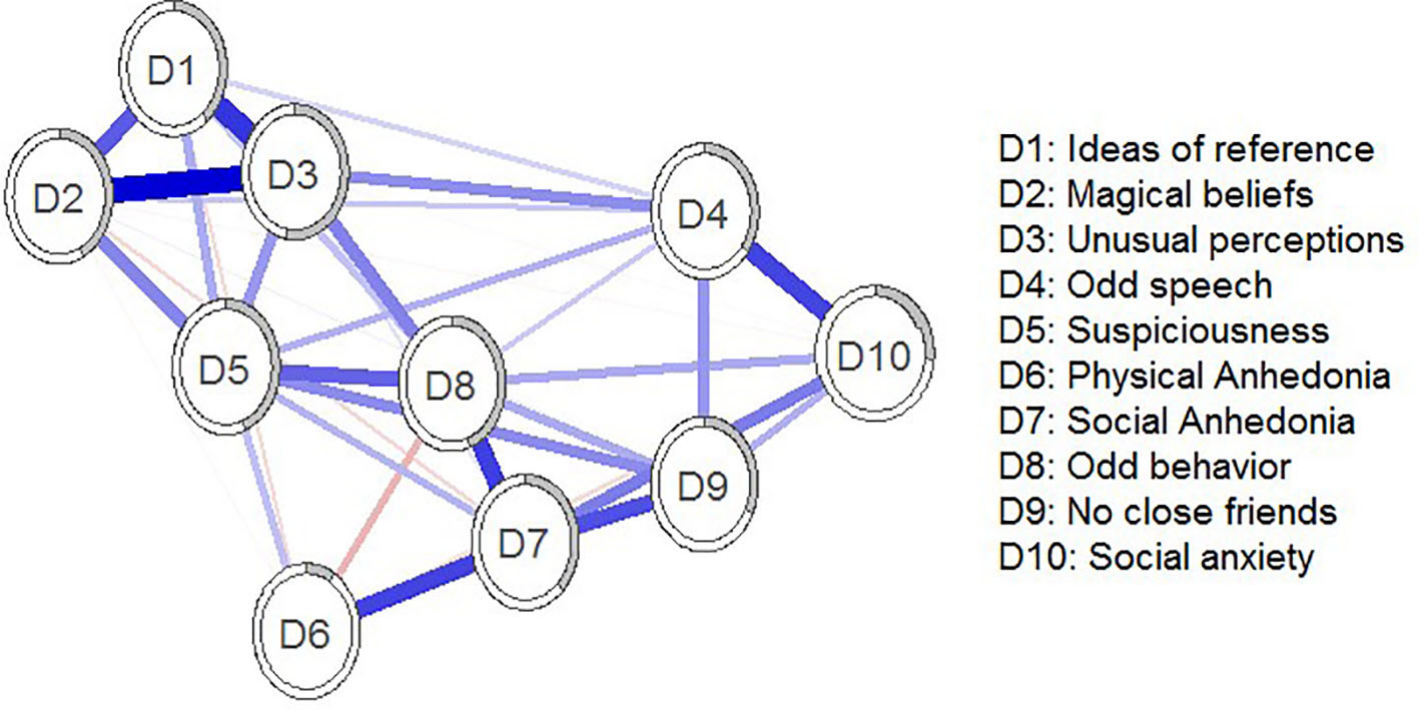
Estimated schizotypy network. Blue edges represent positive associations; red edges represent negative associations. Thickness and saturation of edges indicate the strength of associations. The filled part of the circle around each node shows the predictability of each node, representing the variance of the nodes explained by all nodes with which it is connected.

The estimated schizotypy network was interconnected. Results are shown in **Figure 1**. Strong edges within Positive (‘odd/magical beliefs’, ‘unusual perceptions’, and ‘ideas of reference’), Negative (‘physical anhedonia’ and ‘social anhedonia’), and Disorganization domains (‘no close friends’, ‘constricted affect’, ‘odd behavior’, ‘excessive social anxiety’, and ‘odd speech’) were found.

**Figure 2:**
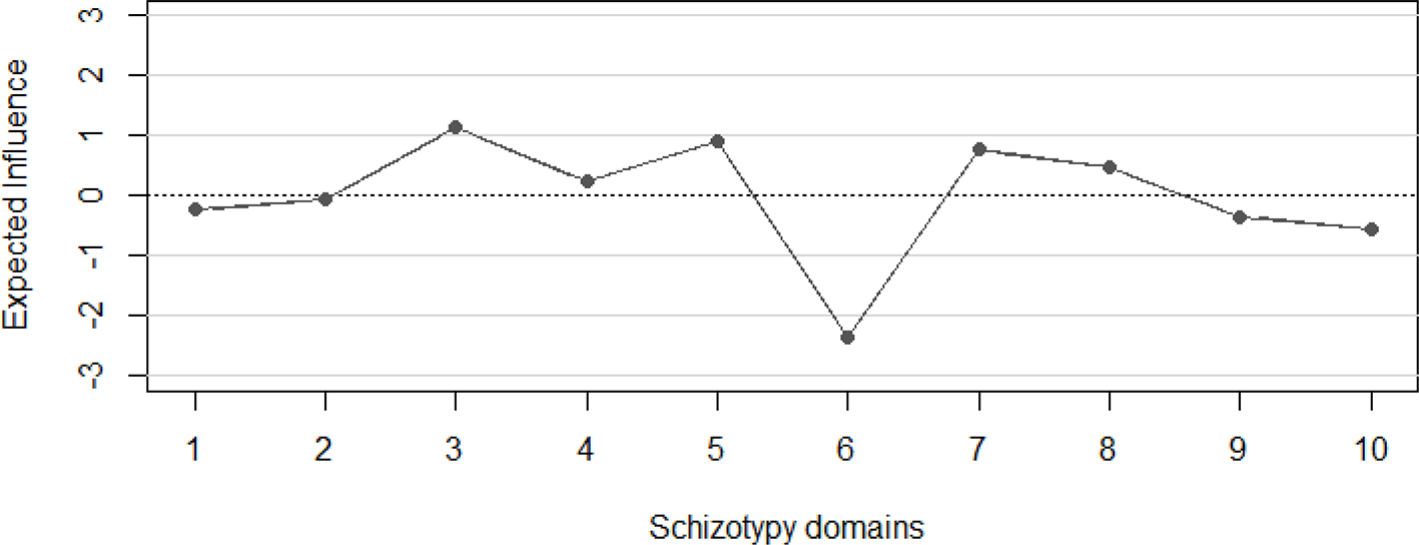
Expected Influence of the domains of the estimated schizotypy network. 1 = Ideas of reference”, 2 = “Magical beliefs”, 3 = “Unusual perceptual experiences”, 4 = “Odd speech”, 5 = “Suspiciousness”, 6 = “Physical Anhedonia”, 7 = “Social Anhedonia”, 8 = “Odd behavior”, 9 = “No close friends”, 10 = “Social anxiety”.

**Figure 2** depicts standardized EI values. The most central nodes in terms of standardized EI were ‘unusual perceptual experiences’ and ‘paranoid ideation’. Predictability ranged from 8.7% (‘physical anhedonia’) to 52.7% (‘unusual perceptual experiences’). The average predictability was 36.27%. The correlation between predictability and EI was 0.92.

### Network Structure of Multidimensional Psychosis Liability

**Figure 3:**
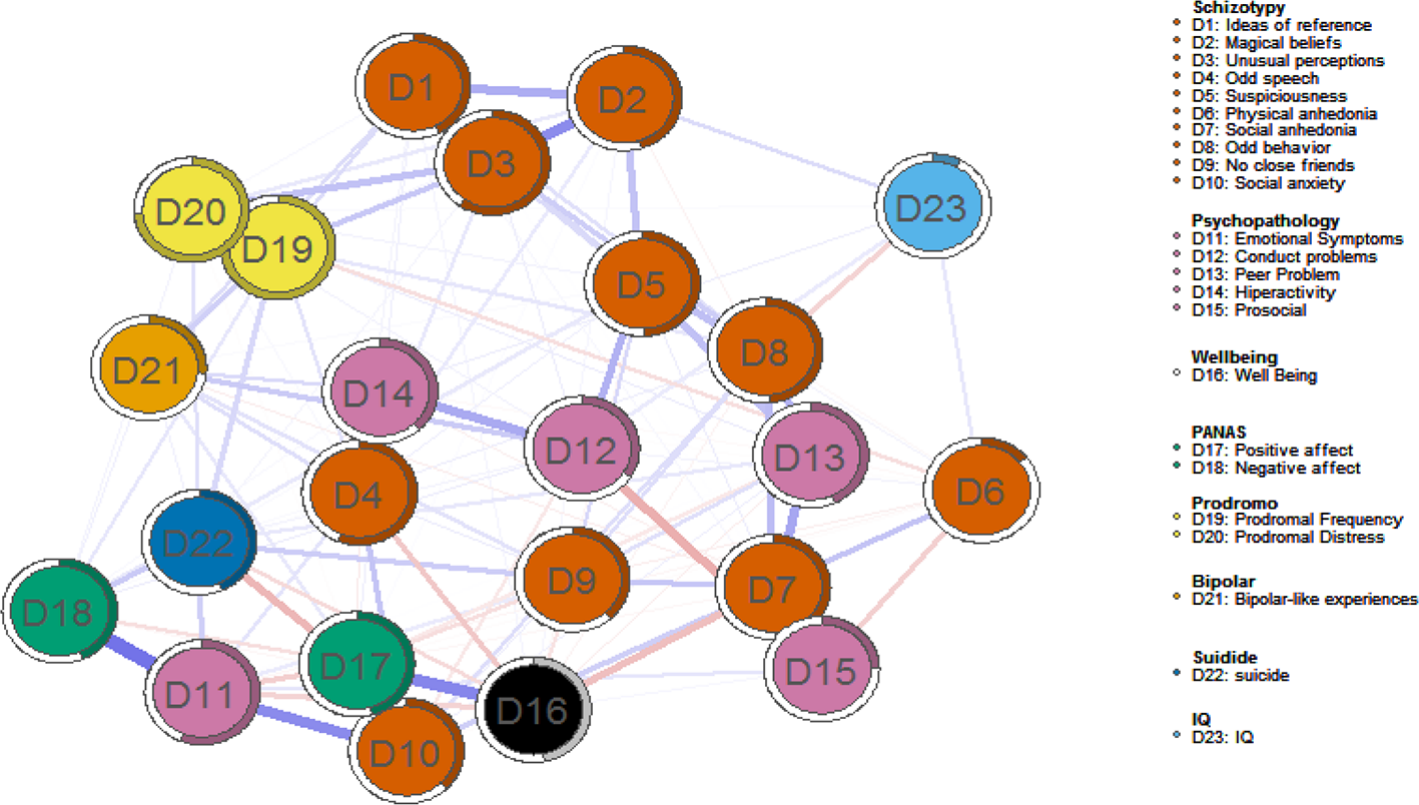
Estimated multidimensional psychosis liability network. Blue edges represent positive associations; red edges represent negative associations. Thickness and saturation of edges indicate the strength of associations. The filled part of the circle around each node shows the predictability of each node, representing the variance of the nodes explained by all nodes with which it is connected.

**Figure 3** shows the estimated network for schizotypy dimensions and related psychopathological, affective, cognitive, and behavioral phenomena. First, strong and positive edges between nodes ‘odd/magical beliefs’, ‘unusual perceptual experiences’, ‘ideas of reference’, ‘suspiciousness’ and ‘psychotic-like experiences’ were found. Second, the majority connections between estimated IQ and other nodes are absent; this implies that these variables can be statistically independent when conditioning on all other nodes, or that there was not sufficient power to detect an edge between these nodes. Third, strong connections emerge among ‘psychotic-like experiences’ and ‘bipolar-like experiences’ nodes. Fourth, protective factors like ‘prosocial behavior’, ‘positive affect’, and ‘subjective well-being’ were positive associated. Especially, strong connections emerge among Node D17 (Positive affect) and Node D16 (personal well-being).

**Figure 4:**
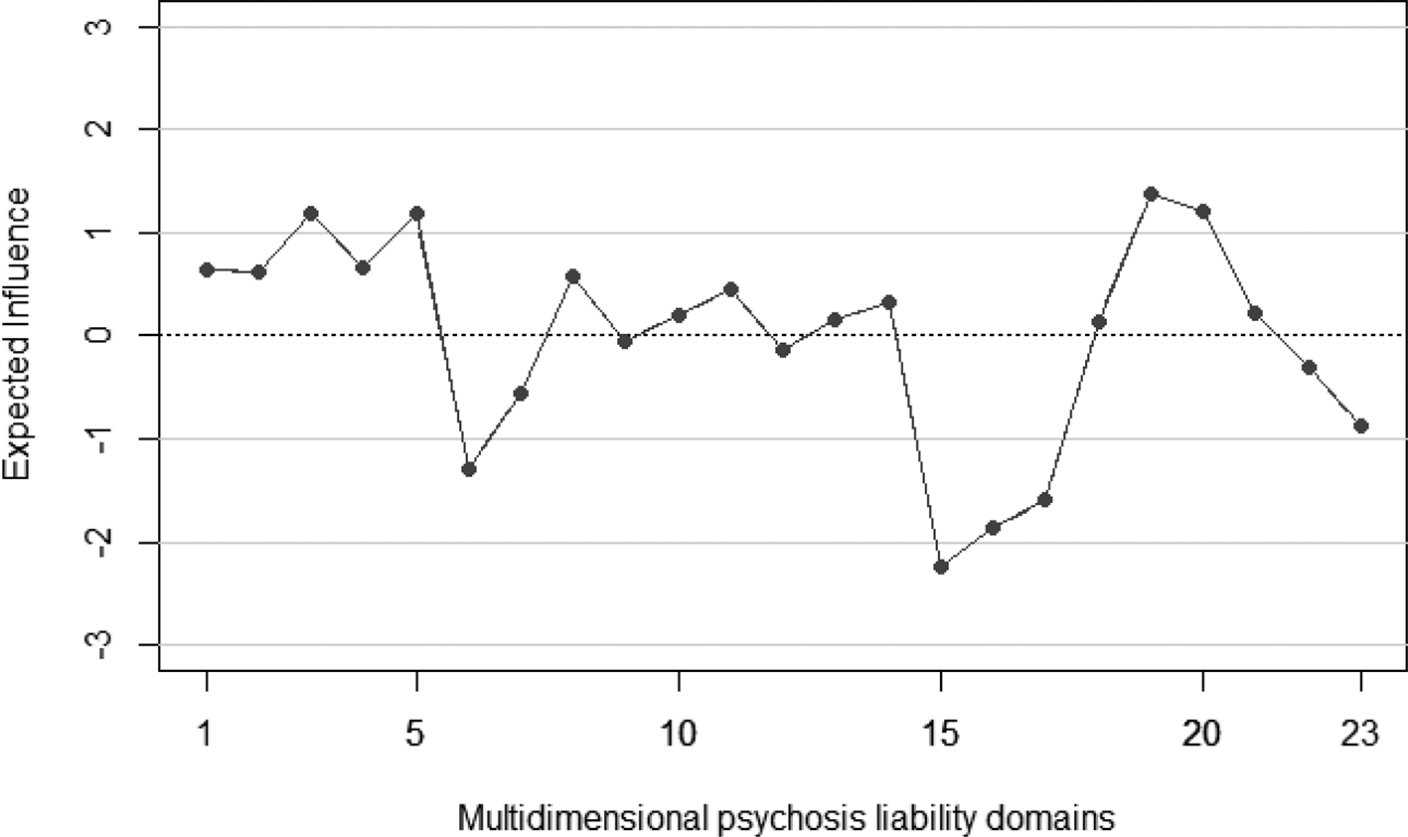
Expected Influence of multidimensional psychosis liability network. 1 = “Ideas of reference”, 2 = “Magical beliefs”, 3 = “Unusual perceptions”, 4 = “Odd speech”, 5 = “Suspiciousness”, 6 = “Physical anhedonia”, 7 = “Social anhedonia”, 8 = “Odd behavior”, 9 = “No close friends”, 10 = “Social anxiety”, 11 = “Emotional Symptoms”, 12 = “Conduct problems”, 13 = “Peer Problem”, 14 = “Hiperactivity”, 15 = “Prosocial”, 16 = “Well Being”, 17 = “Positive affect”, 18 = “Negative affect”, 19 = “Prodromal Frequency”, 20 = “Prodromal Distress”, 21 = “Bipolar-like experiences”, 22 = “Suicide behavior”, 23 = “IQ”.

The most central nodes in terms of standardized EI were ‘unusual perceptions’, ‘suspiciousness’, and ‘psychotic-like experiences’ (both frequency and distress). Results are depicted in **Figure 4**. Interestingly, ‘prosocial behavior’, ‘positive affect’, and ‘subjective well-being’ were the least central domains. Predictability ranged from 9% (estimated IQ) to 74.90% (‘psychotic-like experiences’, both frequency and distress associated). The average predictability was 43.46%. The correlation between predictability and EI was 0.62.

### Network Stability

The results of the stability and accuracy analysis (58) indicated that all networks were accurately estimated. Stability analyses revealed that the networks were accurately estimated, with moderate confidence intervals around the edge weights. The outputs for schizotypy network are presented in the online **Supplemental Materials**.

## Discussion

Here, we proposed to understand schizotypy, a multidimensional psychosis liability index, as a complex system of cognitive, emotional, and behavioral traits. To date, the network structure of schizotypy, as well as its links with other risk and protective indicators, have not been clearly delimited and analyzed. To the best of our knowledge, this is the first study to examine the empirical network structure of schizotypy during adolescence. In addition, no previous studies have examined the multidimensional psychosis liability with a large number of cognitive, affective, behavioral, and social indicators (e.g., mental health difficulties, subjective well-being, bipolar-like experiences, suicide ideation, psychotic-like experiences, positive and negative affect, and IQ). Thus, new approaches, such as network model, may provide new insights in the delimitation and conceptualization of psychosis liability, as well as psychopathology or mental health before clinical outcome and functional impairment. Furthermore, this novel conceptualization, as a complex system, is the first step in embracing the dynamic and complexity of early stages of psychopathology and emerging micro-phenotypes. In addition, this approach might help for the identification, prognosis, prevention, diagnosis, and prophylactic interventions.

The schizotypy domains were strongly interconnected. In particular, the relationship between nodes showed a three-cluster named Cognitive-Perceptual, Interpersonal (Negative), and Disorganized. The average predictability was 36.27%, implying that substantial variability remained unexplained. This network structure found was quite compatible with the three-dimensional model proposed schizotypy/schizotypal research (14, 62, 63). These results are also congruent with previous studies. Network models have also been used to analyze, amongst others, schizotypal personality traits in a multinational sample (54), psychotic like-experiences in cross-cultural study (64), and psychotic-like experiences in a large U.S. sample (65). For instance, Fonseca-Pedrero et al., (54), using the Schizotypal Personality Questionnaire (66), indicated that schizotypal traits were strongly interconnected in the domain-level network. Predictability ranged from 31% (magical thinking) to 55% (restricted affect), with a mean of 43.7%. In another study, Murphy et al. (65) found that psychosis network revealed strong interconnectivity between psychotic-like experiences, where nodes indicating paranoia were among the most central in the estimated network. In addition, the viewpoint of psychosis phenotype, as a network system, is congruent with previous research that demonstrated how negative/disorganized symptoms predicted positive symptoms (67) or how hallucinations gave rise to delusions (68).

The network structure between schizotypy, mental health difficulties, subjective well-being, bipolar-like experiences, suicide ideation and behavior, psychotic-like experiences, positive and negative affect, prosocial behavior, and estimated IQ was analyzed. Variables showed relations both within and across domains, although within-domain associations were generally stronger. The network predictability values ranged from 9% (estimated IQ) to 74.90% (‘psychotic-like experiences’), where the mean value of predictability was 43.46%. The psychosis-like experiences in terms of frequency and distress associated were the most central nodes in this estimated network. Also, suicide ideation and behavior were connected to negative affect and psychotic-like experiences. These results are consistent with previous studies conducted in other samples and with other measuring instruments (69–71). For instance, Zhang et al. (71) investigated the network structure between schizotypal traits and autistic traits, obsessive-compulsive traits, depressive symptoms, and anxiety symptoms in a college sample. They found that schizotypal features were highly overlap with depressive symptoms, however anxiety symptoms only connected with interpersonal traits. In addition, the network estimated showed high predictability, similar to the value yielded in the present study, where interpersonal traits had the highest expected influence. Interestingly, beyond to traditional psychopathology viewpoint, protective factors like prosocial behavior, positive affect, and subjective well-being were, on the one hand, more closely associated with each other than with other dimensions and, on the other hand, negative related with psychosis liability dimensions (e.g., ‘ideas of reference’, ‘unusual perceptual experiences’) and mental health difficulties (e.g., peer problems, emotional symptoms). To date, no previous studies have analyzed the psychosis liability network using both risk and protective factors. In this sense, it is plausible to argue that good subjective quality of life, positive emotions, or prosocial conduct might act as protective factors, leading to more resilient networks and becoming a less interconnected symptom network (22). This estimated network might be an example of the emerging psychopathology as a mixture picture of affective dysregulation, aberrant salience, cognitive impairments, and behavioral difficulties. Future studies should analyze the role of protective factors in psychosis extended phenotype as key elements to promoting well-being in young people, whether at risk or not.

Another relevant point in the present research is the role played by the estimated IQ in the multidimensional psychosis liability network. In the overall network, the associations between IQ and other nodes were generally low. Nonetheless, several issues have to be mentioned. First, IQ was measured by only a short task of complex reasoning (i.e., matrices test). Second, IQ was measured by an objective task while other indicators where measured by self-report tools. Third, adolescence is a developmental stage where executive and cognitive functions may develop at different pace. Fourth, the data were recollected both from different levels of analyses and measured with different tools. These facts might affect to the results found. However, we have to recognize that IQ (by extension cognitive abilities) is a key factor in the psychosis picture (both clinical and subclinical). Previous studies have demonstrated that people with psychosis have deficits in a wide variety of cognitive domains, in particular intelligence (72). In addition, such deficits are present in the premorbid stage and in the prodromal or at-risk mental phase, and predict the emergence of full-blown psychosis (73, 74). Therefore, to real understanding the psychosis liability it is relevant to gather information of IQ, because it is a multidimensional phenotype that requires cognitive, affective, psychophysiological, social, and behavioral variables. In addition, it is possible that accessing and analyzing data on multiple indicators, simultaneously, and from several levels of analyses, might accelerate the prediction of disease progression, as well as contribute to a better understanding of etiological mechanisms. To date, no previous studies have examined the network multidimensional structure of psychosis liability using IQ estimators. Thus, future studies in this line are still necessary.

These findings are congruent with the idea of transdiagnostic psychosis spectrum encompassing both non-affective and affective psychotic experiences (7) as well as with the psychosis proneness-persistence-impairment model (75). In particular, this model posits that the developmental expression of psychosis may become abnormally persistent and subsequently clinically relevant if there is a combination of other genetic, environmental, and psychological factors (7, 12). Thus, the presence of schizotypal traits or subclinical psychotic symptoms is not a necessary or sufficient condition for the later development of a psychotic disorder or other mental disorders (10, 12). Worth noting, the psychosis liability may interact synergistically or additively with genetic (e.g., unaffected family members of patients with psychosis), environmental (e.g., trauma, cannabis use), and/or psychological factors (e.g., affective dysregulation, avoidance coping). In addtion, this latent liability could causally impact on each other over time in a network of dynamic interactions, becoming abnormally persistent, help-seeking, and eventually give rise to transition to a psychotic spectrum disorder and impairment (12, 30, 7). For instance, Isvoranu et al. (76) demonstrated that psychosis symptom networks were more strongly connected for people exposed to environmental risk factors (e.g., cannabis use, developmental trauma, urban environment), indicating that environmental exposure may lead to a more strongly connected network structure and less resilient symptom networks. As Lenzenweger (2) pointed out, mental disorders represent complex configural outcomes of multiple interacting systems that cannot be reduced to a mere collection of constituent parts.

Some limitations of this study should be acknowledged. First, adolescence is a developmental period in which brain, cognition, and personality are still consolidating. Second, in the present study, we only investigated the schizotypy through self-report screening measures. These measures have been associated with stigmatization and negative labeling. Third, it should be borne in mind that this study was of a cross-sectional nature, so we cannot make cause-effect inferences. Fourth, the results found in the present study needs longitudinal confirmation. Fifth, regarding the structure of the estimated network, a correct interpretation of it should not only focus on the visual inspection of its topography. A problem to avoid in the estimated networks, is precisely the over-interpretation on its visualization (77). This aspect refers especially to the design and placement of nodes in the graph, for example, when the nodes of the network are grouped in a cluster. However, it is relevant to know that the location of the node within a network is only one of the many equally ‘correct’ ways of placing the nodes in it, that is, with the same one showing the distribution of the nodes in the network. This network, in a new estimate, could be different. Also, the fact that a node is at the center of the network does not necessarily indicate that it is the most “central” node in it. We must be cautious when making a visual interpretation of the nodes and the analysis of their importance depending on the position in the estimated network. Therefore, for a better interpretation of the psychological network, and in order to avoid incorrect inferences, it is relevant to use other indicators as: predictability (78) or other statistical procedures (77). Finally, research in network analysis is currently in its infancy, and is not free of tentative limitations (e.g., generalizability and reproducibility of network estimation) (79, 80), so it is necessary to continue working on the construction of a solid and refutable scientific model and to incorporate new scientific evidence (22).

## Conclusions

This study is the first to comprehensively examine the network structure of schizotypy, as an indicator of psychosis liability, using a large sample of adolescents. The results are consistent with the conceptual notion of schizotypy, understood as a complex network structure of cognitive, emotional, and behavioral traits. This study also offers a deeper understanding of the subclinical psychosis expression (psychosis liability) and its links with psychopathology, affective, personality, and cognitive domains. The understanding of the network structures of psychosis liability in general population may help to prevent psychotic-spectrum and mental health disorders. Finally, network analyses represent a data-driven approach allowing the investigation of the complex relationships of psychosis liability expressions and processes, including not only risk factors but also protective factors. Future studies should incorporate different scale levels of observation, like environmental and genetic variables, into network models.

## Data Availability Statement

The datasets generated for this study are available on request to the corresponding author.

## Ethics Statement

The research was approved by the Educational Government of La Rioja and the Ethical Committee of Clinical Research of La Rioja (CEICLAR). For participants under 18, parents were asked to provide a written informed consent in order for their child to participate in the study. Participants were informed of the confidentiality of their responses and of the voluntary nature of the study.

## Author Contributions

EF-P designed the research and contributed with data analysis, and text writing. JO-S contributed with data analysis and text writing. FI contributed with text writing. JR-T contributed with text writing and helped in the design of the research. MD contributed with data analysis and text writing.

## Funding

EF-P was supported by la Convocatoria 2015 de “Ayudas Fundación BBVA a Investigadores y Creadores Culturales”, by “Ayudas Fundación BBVA a equipos de investigación científica 2017”, and FEDER La Rioja 2014-2020 (SRS 6FRSABC026). MD was supported by a grant from the Swiss National Science Foundation (100019_159440).

## Conflict of Interest

The authors declare that the research was conducted in the absence of any commercial or financial relationships that could be construed as a potential conflict of interest.
