# Supplementary material for: Beyond Clinical High-Risk State for Psychosis: The Network Structure of Multidimensional Psychosis Liability in Adolescents
Source: Front Psychiatry. 2020 Feb 11;10:967. doi: 10.3389/fpsyt.2019.00967 (PMC7026502; doi:10.3389/fpsyt.2019.00967)
Supplement: Supplementary file 1 [file DataSheet_1.docx]

Table S1. Pearson's correlations between measures.

|  | 1 | 2 | 3 | 4 | 5 | 6 | 7 | 8 | 9 | 10 | 11 | 12 | 13 | 14 | 15 | 16 | 17 | 18 | 19 | 20 | 21 |
| --- | --- | --- | --- | --- | --- | --- | --- | --- | --- | --- | --- | --- | --- | --- | --- | --- | --- | --- | --- | --- | --- |
| Prodromol frequency (1) |  |  |  |  |  |  |  |  |  |  |  |  |  |  |  |  |  |  |  |  |  |
| Prodromal distress (2) | .837** |  |  |  |  |  |  |  |  |  |  |  |  |  |  |  |  |  |  |  |  |
| Suicide behaviour (3) | .439** | .461** |  |  |  |  |  |  |  |  |  |  |  |  |  |  |  |  |  |  |  |
| Positive Affect (4) | -.289** | -.339** | -.512** |  |  |  |  |  |  |  |  |  |  |  |  |  |  |  |  |  |  |
| Negative Affect (5) | .432** | .422** | .424** | -.411** |  |  |  |  |  |  |  |  |  |  |  |  |  |  |  |  |  |
| Subjective well-being (6) | -.309** | -.310** | -.441** | .558** | -.380** |  |  |  |  |  |  |  |  |  |  |  |  |  |  |  |  |
| Bipolar-like experiences (7) | .388** | .320** | .204** | -.079** | .253** | -.116** |  |  |  |  |  |  |  |  |  |  |  |  |  |  |  |
| Emotional problems (8) | .435** | .460** | .462** | -.480** | .599** | -.469** | .196** |  |  |  |  |  |  |  |  |  |  |  |  |  |  |
| Behaviour problems (9) | .312** | .312** | .241** | -.164** | .263** | -.187** | .308** | .175** |  |  |  |  |  |  |  |  |  |  |  |  |  |
| Peer problems (10) | .358** | .385** | .379** | -.361** | .273** | -.392** | .142** | .347** | .238** |  |  |  |  |  |  |  |  |  |  |  |  |
| Hiperactivity (11) | .288** | .264** | .178** | -.151** | .250** | -.179** | .299** | .222** | .367** | .116** |  |  |  |  |  |  |  |  |  |  |  |
| Prosocial Behaviour (12) | -.087** | -.097** | -.112** | .139** | -.038 | .196** | -.066* | -0.031 | -.293** | -.185** | -.110** |  |  |  |  |  |  |  |  |  |  |
| Ideas of reference (13) | .494** | .462** | .223** | -.107** | .244** | -.145** | .313** | .225** | .245** | .239** | .160** | -0.028 |  |  |  |  |  |  |  |  |  |
| Magical thinking (14) | .473** | .466** | .267** | -.140** | .271** | -.161** | .226** | .270** | .239** | .193** | .203** | -.065* | .508** |  |  |  |  |  |  |  |  |
| Unusual perceptual exp. (15) | .631** | .619** | .397** | -.229** | .306** | -.266** | .304** | .342** | .317** | .324** | .247** | -.137** | .560** | .592** |  |  |  |  |  |  |  |
| Odd speech (16) | .476** | .452** | .343** | -.368** | .370** | -.440** | .343** | .448** | .317** | .267** | .554** | -.154** | .345** | .374** | .446** |  |  |  |  |  |  |
| Paranoid ideation (17) | .464** | .461** | .362** | -.298** | .357** | -.362** | .287** | .340** | .405** | .468** | .227** | -.196** | .428** | .458** | .511** | .412** |  |  |  |  |  |
| Physical anhedonia (18) | -.134** | -.087** | -.017 | -.046 | -.057* | -.071** | -.074** | -.071** | .089** | -.019 | .021 | -.203** | -.054* | -.042 | -.026 | -.009 | .053* |  |  |  |  |
| Social anhedonia (19) | .274** | .281** | .314** | -.349** | .210** | -.447** | .075** | .267** | .205** | .488** | .086** | -.406** | .218** | .182** | .300** | .293** | .383** | .195** |  |  |  |
| Odd behaviour (20) | .463** | .420** | .341** | -.286** | .280** | -.382** | .233** | .324** | .258** | .497** | .186** | -.182** | .392** | .355** | .485** | .401** | .513** | -.051* | .463** |  |  |
| Lack of close friends (21) | .347** | .343** | .405** | -.404** | .309** | -.427** | .262** | .402** | .245** | .415** | .172** | -.173** | .254** | .265** | .306** | .378** | .417** | -.044 | .432** | .424** |  |
| Social anxiety | .321** | .300** | .233** | -.277** | .318** | -.349** | .118** | .508** | .074** | .330** | .152** | -.104** | .199** | .182** | .273** | .413** | .280** | -.027 | .369** | .367** | .338** |

Note. **p*<.05; ***p*<.01

Figure S1. Bootstrapped edge-weights for the total sample. Schizotypy Network. The red line depicts point estimates of the edge weights, the grey bar 95% confidence intervals.


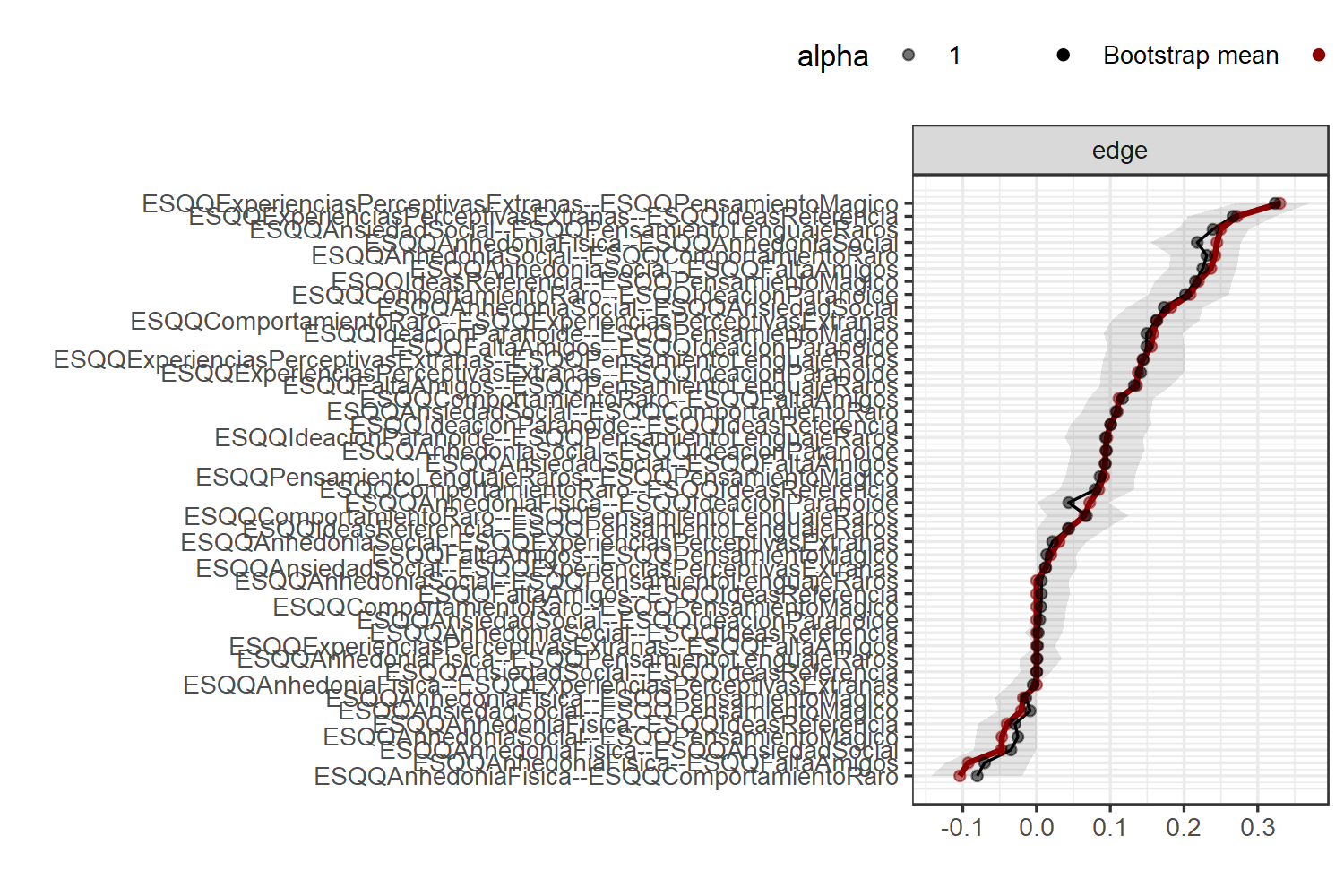


Figure S2. The correlation between the original centrality index and the centrality index after dropping a percentage of subjects at random from the data in the full dataset. Stability centrality coefficients (i.e. % of cases that can be dropped to retain with 95% certainty a correlation of 0.7 of centrality between network estimated on original data and network estimated on subsampled data). Schizotypy network.


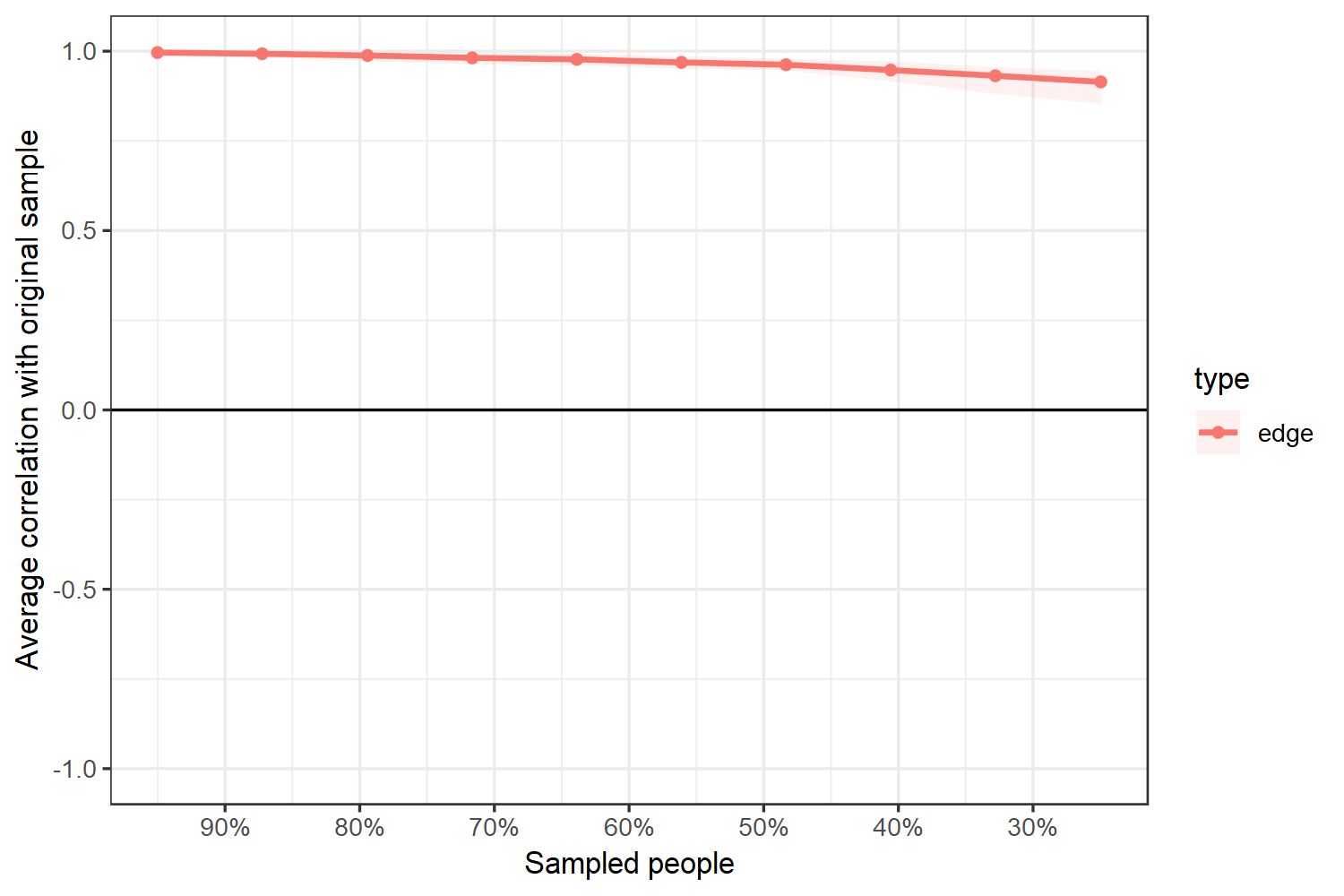


Figure S3. Network thickness interpretation.


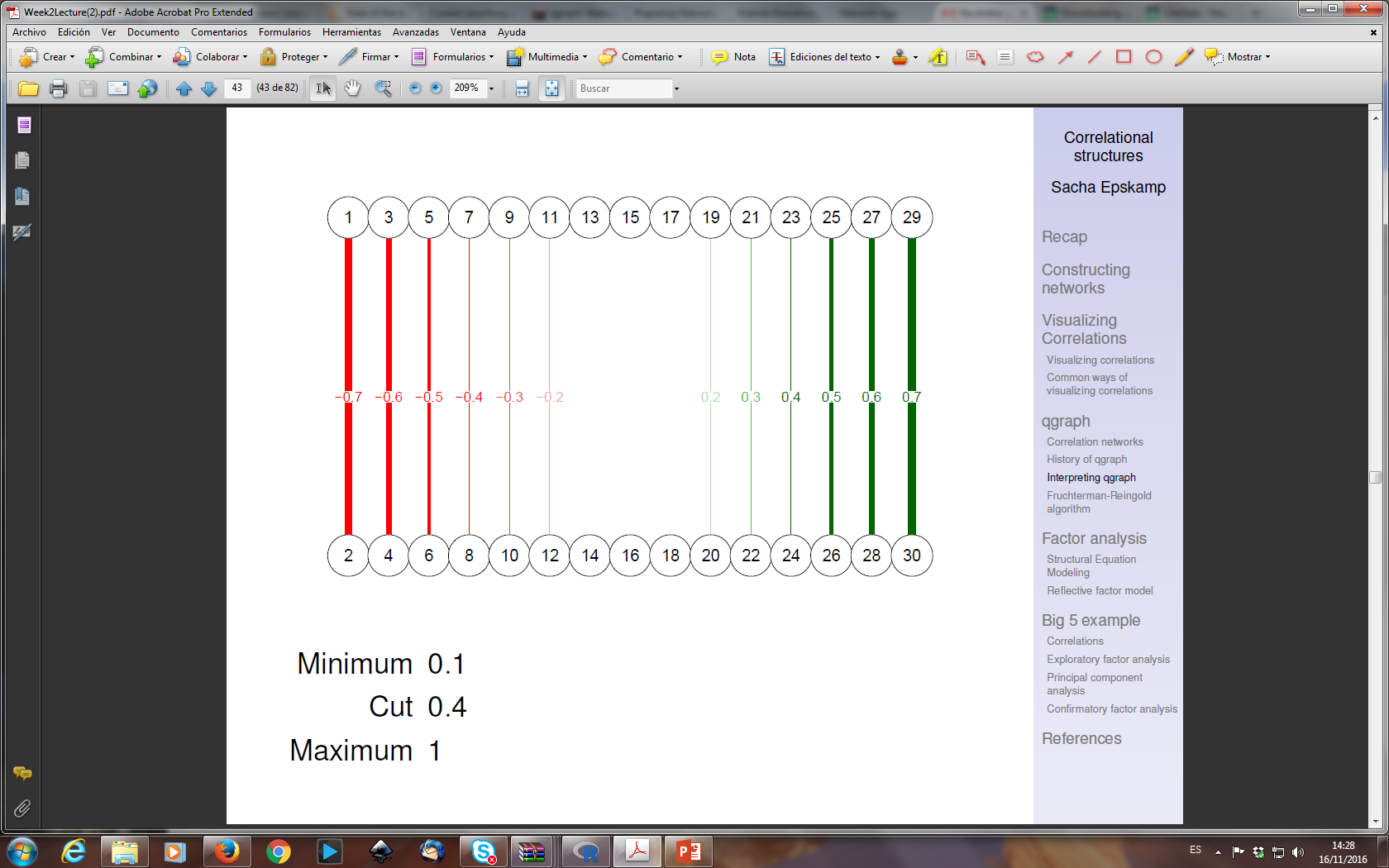


Note. To interpret *qgraph* networks, three values need to be known: a) Minimum Edges with absolute weights under this value are omitted. Maximum If set, edge width and colour scale such that an edge with this value would be the widest and most colourful. Cut If specified, splits scaling of width and colour. Figure download from: http://sachaepskamp.com/.
